# Melting, Crystallization, and In Vitro Digestion Properties of Fats Containing Stearoyl-Rich Triacylglycerols

**DOI:** 10.3390/molecules27010191

**Published:** 2021-12-29

**Authors:** Kwang-Seup Shin, Jeung-Hee Lee

**Affiliations:** Department of Food and Nutrition, Daegu University, Gyeongsan 38453, Korea; botomak2@naver.com

**Keywords:** stearoyl-rich fats, melting, crystallization, digestibility, 1,2-distearoyl-3-oleoylglycerol, 1,3-dioleoyl-2-stearoylglycerol

## Abstract

Fats containing the stearoyl-rich triacylglycerols (TAGs) of 1,2-distearoyl-3-oleoylglycerol (SSO) and 1,3-dioleoyl-2-stearoylglycerol (OSO) were synthesized via the lipase-catalyzed acidolysis of tristearin (SSS)-rich fat and oleic acids, followed by solvent fractionation. Their physicochemical properties and in vitro digestibilities were compared. The SSS-, SSO-, and OSO-rich fats comprised 81.6%, 52.9%, and 33.1% stearic acid, respectively, whereas oleic acid comprised 2.9%, 37.5%, and 56.2%, respectively. The SSS-, SSO-, and OSO-rich fats contained the TAGs of SaSaSa (100.00%), SaSaMo (86.98%), and MoSaMo (67.12%), respectively, and the major TAGs were SSS, SSO, and OSO, respectively. Melting and crystallization temperatures were higher and fat crystals were larger and densely packed in the descending order of SSS-, SSO and OSO-rich fats. Both in vitro multi-step digestion and pH-stat digestion were more rapid for OSO- than SSO-rich fat. Oleic acid was digested faster than stearic acid during the initial digestion, then the rate decreased, whereas that of stearic acid increased over prolonged digestion. Fats that were richer in stearoyl at the *sn*-1,3 position of TAG melted and crystallized at higher temperatures, had a densely packed microstructure of large fat crystals and were poorly digested. Stearic acid imparts the essential physical attributes of melting and crystallization in solid fats, and the low digestible stearoyl-rich fat would be a viable substitute for trans fatty acids in food lipid industry.

## 1. Introduction

The type and stereospecificity of fatty acids (FAs) in triacylglycerols (TAGs) determine the physicochemical and digestion properties of dietary fats, oils, and food products. Generally, TAGs have a saturated FA such as palmitic (P) or stearic (S) acid at the *sn*-1 (or 3) position and an unsaturated FA such as oleic acid (O) or linoleic (L) acid at the *sn*-2 position. They comprise the major TAGs of POS, SOS, and POP in cocoa butter, POO, POP, and POS in beef tallow, and SPO, OPL, and OPO in lard [1]. The physicochemical properties of fats, such as TAG profiles, crystallization behavior, solid fat content, and melting point, depend on the incorporated FAs that influence the quality of sensory characteristics such as ‘mouth feel’ in chocolate or ice-cream and ‘lightness’ in pastry [1]. 

The digestion, absorption, and metabolic fate of dietary fats are determined by the stereospecificity (*sn*-1,3 or *sn*-2) and FAs with different chain lengths and degrees of saturation/unsaturation in TAG molecules [2]. TAGs are generally hydrolyzed in the duodenum by pancreatic lipase, which has greater affinity for ester bonds in the *sn*-1 (or 3) position of TAG, resulting in the release of free FAs (FFAs) and 2-monoacylglycerol (2-MAG), in which most FAs at the *sn*-1 (or 3) positions are hydrolyzed, compared with 22% of those in the *sn*-2 position [3]. Pancreatic lipase is more active against short- than long-chain FAs and against unsaturated than saturated FAs [2]. FAs with chain lengths >12 carbons at the *sn*-1 (or 3) positions are absorbed in intestinal mucosal cells where they are reassembled into new TAGs that are transported in chylomicrons to the lymph and finally into the circulation. Short (C4–C6) and medium (C8–C12) chain FAs are directly absorbed into the portal system. Fatty acids at the *sn*-2 position of ingested TAGs are preserved and absorbed intact [4]. However, long-chain saturated FAs such as stearic acid in the *sn*-1 (or 3) position are hydrolyzed, bound to calcium or magnesium ions, then excreted as an insoluble FA soap [1,2].

Saturated FAs generally increase serum total cholesterol (TC) [5]. Regression equations developed to predict the relationship between blood cholesterol concentrations and dietary FAs described in 18 published articles have shown that stearic acid does not affect plasma TC, low-density lipoprotein cholesterol (LDL-C), and high-density lipoprotein cholesterol (HDL-C) levels, whereas the saturated FAs such as myristic, lauric, and palmitic acids significantly increases them [6]. A meta-analysis of 60 controlled trials has shown that dietary stearic acid reduces the ratio of TC/HDL-C, which is an estimated risk factor for cardiovascular disease (CVD), whereas palmitic acid has little effect on this ratio [7]. The atherogenic index is calculated with lauric acid, myristic acid, palmitic acid, trans fatty acids (TFAs), and unsaturated fatty acids, and the higher the content of TFAs and myristic acid, the higher the atherogenic index [8]. Compared with TFAs, dietary stearic acid significantly increases HDL-C and apolipoprotein (apo) A-I, and decreases LDL-C and apo B, but does not affect the LDL-C/HDL-C ratio and apoB/apoA-I, whereas TFA increases these ratios [9]. Stearic acid has the requisite physical attributes of a solid fat, and it is being considered as a viable substitute for TFAs in food manufacturing, as well as an indirect contributor to health because TFA is a risk factor for CVD, and the recommended dietary intake of TFA is as low as possible [10]. 

TFAs are formed as by-product during hydrogenation which is a process to convert liquid oil to semisolid fats for usage of shortening and margarine in food lipid industry [11]. For alternatives to hydrogenated types of bakery shortening, *trans*-free bakery fat was produced by lipase-catalyzed interesterification with fully hydrogenated soybean oil (FHSBO), rapeseed oil, and palm stearin, and this fat was reported to contain the desirable β′ form of crystal and had suitable melting and crystallization behaviors including solid fat content [12]. Zero-*trans* shortening fat synthesized with FHSBO and high oleic sunflower oil through a lipase-catalyzed interesterification, comprised the major TAGs of OSO/SOO (36.9%) and SSO/SOS (34.4%), and showed the desirable properties of bakery fat [13]. 

The physicochemical properties of fats specifically modified to have high content or rearranged positions of stearic acid on TAGs produced by interesterification and/or solvent fractionation, have been investigated. Asymmetric 1,2-distearoyl-3-oleoylglycerol enriched lipid has been synthesized by lipase-catalyzed interesterification of FHSBO and oleic ethyl ester, and its application as a cocoa butter replacer (CBR) has been evaluated based on its melting characteristics [14]. Lipase-catalyzed fat as a substitute for zero-*trans* shortening fat has been synthesized using a high oleic sunflower oil and FHSBO, and its melting point, solid fat content, and melting behavior have been determined [13]. In addition to these physicochemical properties, the modified fat had different digestibility and absorption characteristics. For example, the synthetic fat substitute, SALATRM, is a structured TAG containing predominantly stearic acid and short-chain acetic, propionic, and butyric acids. Stearic acid excretion from this substitute is increased, which leads to low digestibility [15]. 

The demand for stearic acid is increasing as a substitute for *trans*-fats in food lipids. Therefore, fats rich in SSO and OSO were synthesized via lipase-catalyzed acidolysis with SSS-rich fat (as FHSBO) and oleic acid-rich fatty acids. Then, their physicochemical characteristics and digestibility were evaluated according to the concentrations and stereospecific positions of stearic acid. The physicochemical properties of SSS-, SSO-, and OSO-rich fats were compared by analyzing the composition of acylglycerols, TAGs, and FAs, melting and crystallization behaviors, solid fat index, and crystal morphology. The digestibility property of SSO-rich and OSO-rich fats was compared by analyzing hydrolysis rates and the hydrolyzed FFA profile using in vitro pH-stat model and multi-step digestion model.

## 2. Results and Discussion

### 2.1. Fatty Acid Composition

The major FAs in SSS-rich fat were stearic (C18:0, 81.63%), palmitic (C16:0, 13.09%), and oleic (C18:1, 2.86%) acids, and the main FAs at the *sn*-2 position were stearic (82.69%), oleic (9.64%), and palmitic (4.75%) acids, whereas those at *sn*-1,3 were stearic (80.66%) and palmitic (17.24%) acids (Table 1 and Table 2). Oleic acid-rich FAs mainly consisted of oleic acid (90.53%) and small amounts of stearic, elaidic (C18:1*t*), and linoleic (C18:2) acids. The FAs of SSO-rich and OSO-rich fats consisted of stearic, oleic, palmitic, and linoleic acids, and the stearic acid content was significantly higher in SSO-rich fat (52.89%) than in OSO-rich fat (33.14%) (*p* < 0.05), whereas the oleic acid content was higher in OSO-rich fat (65.23%) than in SSO-rich fat (37.45%) (*p* < 0.05) (Table 1). The *sn*-2 position contained 1.12-fold more stearic acid in SSO-rich fat (94.39%) than in OSO-rich fat (83.74%), and 4.04-fold more oleic acid in OSO-rich fat (11.96%) than in SSO-rich fat (2.96%) (*p* < 0.05). The *sn*-1,3 position contained 1.43-fold more oleic acid in OSO-rich fat (78.36%) than in SSO-rich fat (54.70%) (*p* < 0.05) and 4.1-fold more stearic acid in SSO-rich fat (32.14%) than in OSO-rich fat (7.84%) (*p* < 0.05) (Table 2). Lipozyme^®^ RMIM is a lipase that selectively hydrolyzes the ester bond at the *sn*-1 and *sn*-3 positions of TAGs but not at the *sn*-2 position. Lipozyme RMIM catalyzed the acidolysis reaction between substrates of SSS-rich fat and oleic acid rich FFAs, where FAs (mostly stearic acid and palmitic acid) at the *sn*-1(3) positions of TAGs in SSS-rich fat were hydrolyzed, whereas oleic and stearic acids were simultaneously re-esterified in the TAGs producing SSO- and OSO-rich fats. The SSO- and OSO-rich fats both consisted of 90.34% and 89.37% stearic and oleic acids, respectively. The positions of the stearic and oleic acids on the glycerol backbones of the TAG molecules distinctly differed, indicating more abundant incorporation of stearic and oleic acids into SSO- and OSO-rich fats, respectively.

Nutritional quality of lipid is defined with the FA composition by calculating the atherogenic index (AI) which is used to assess the risk of cardiovascular diseases. The AI is calculated with three highly atherogenic fatty acids (lauric, myristic, and palmitic acids), TFAs, and unsaturated fatty acids, and the higher the content of TFAs and myristic acids, the higher the AI [8]. The TFAs, not stearic acid, are used for the equation formula due to their high arteriosclerosis potential. The calculated AIs of SSO-rich and OSO-rich fats were 0.16 and 0.10, respectively, and which were much lower than those of margarines (0.23~1.67) [16]. 

### 2.2. Acylglycerol and TAG Composition

The acylglycerols of SSS-rich fat consisted mainly of TAGs (98.31 mmol%), and small amounts of diacylglycerol (DAG) (1.38 mmol%) containing more 1,3-DAG than 1,2-DAG and monoacylglycerol (MAG) (0.31 mmol%) containing more 1-MAG than 2-MAG (Table 3). The acylglycerols of SSO- and OSO-rich fats consisted of mostly TAG (94.68–98.58 mmol%), and a small amount of DAG (0.97–4.78 mmol%) that contained more 1,3-DAG (0.55–3.65 mmol%) than 1,2-DAG (0.42–1.13 mmol%), and MAG (0.45–0.53 mmol%), which contained more 1-MAG (0.24–0.33 mmol%) than 2-MAG (0.20–0.21 mmol%) (Table 3). 

**Figure 1:**
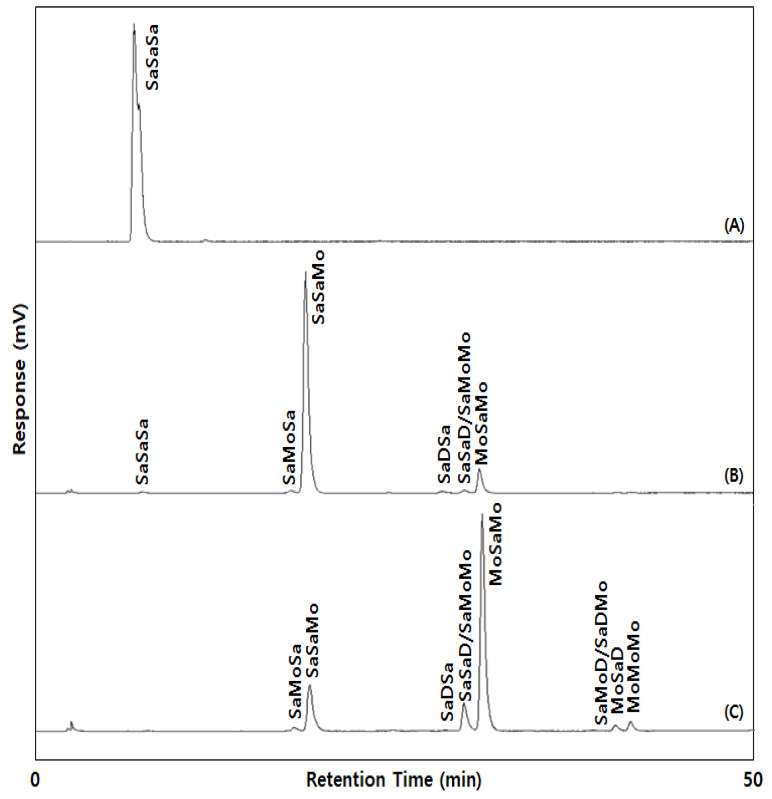
The Ag-HPLC chromatograms of SSS-rich (**A**), SSO-rich (**B**), and OSO-rich (**C**) fats. SaSaSa (SSS, PPP, SSP, PSP, PPS, SPS; major TAG: SSS), SaMoSa (SOS, POS, POP), SaSaMo (SSO, PPO, PSO, SPO; major TAG: SSO), SaDSa (SLS, PLP, SLP), SaSaD/SaMoMo (SSL, PPL, PSL, SPL/SOO, POO), MoSaMo (OSO, OPO; major TAG: OSO), SaDMo/SaMoD (SLO, PLO/SOL, POL), MoSaD (OSL, OPL), MoMoMo(OOO); P: palmitic acid, S: stearic acid, O: oleic acid, L: linoleic acid, Sa: saturated fatty acid, Mo: monounsaturated fatty acid, D: diunsaturated fatty acid.

The TAGs in the fats were identified by TAG separation with silver ion-HPLC (Figure 1) and the FA composition determined with GC (Table 1 and Table 2). The TAGs were identified as SaSaSa (SSS, PPP, SSP, PSP, PPS, and SPS), SaMoSa (SOS, POS, and POP), SaSaMo (SSO, PPO, PSO, and SPO), SaDSa (SLS, PLP, and SLP), SaSaD/SaMoMo (SSL, PPL, PSL, SPL/SOO, and POO), MoSaMo (OSO and OPO), SaDMo/SaMoD (SLO, PLO/SOL, and POL), MoSaD (OSL and OPL), and MoMoMo (OOO; Table 4). The SSS-rich fat consisted mainly of SaSaSa, the major TAG being SSS. The SSO-rich fat consisted of TAGs in SaSaMo (major TAG: SSO, 86.98%) and MoSaMo (major TAG: OSO, 9.21%), whereas the OSO-rich fat consisted of TAGs in MoSaMo (major TAG: OSO, 67.17%), SaSaMo (major TAG: SSO, 17.22%), and SaSaD/SaMoMo (8.55%).

### 2.3. Melting and Crystallization Behaviors 

The slip melting points (SMPs) of SSS-, SSO-, and OSO-rich fats were 69.75 °C, 32.50 °C, and 19.75 °C, respectively, and the complete melting points (CMPs) were 71.25 °C, 38.75 °C, and 24.25 °C, respectively (Table 1). The SMP and CMP were significantly higher for SSS-rich fat than SSO- and OSO-rich fat (*p* < 0.05) because the SSS-rich fat comprised trisaturated TAGs (SSS, PSS, and PSP) with high melting points, whereas the SSO- and OSO-rich fats comprised mainly desaturated (SaSaMo) and monosaturated (MoSaMo) TAGs, respectively. The melting point of fat is influenced by the type of the constituent FAs, and a higher SFA content results in a higher melting point. 

**Figure 2:**
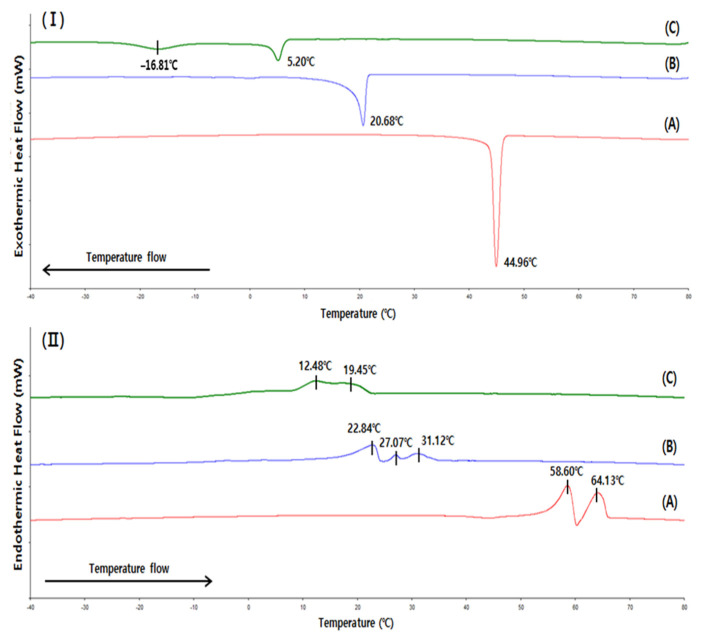
Differential scanning calorimetry (DSC) crystallization (**I**) and melting (**II**) curves of SSS-rich fat (**A**), SSO-rich fat (**B**) and OSO-rich fat (**C**).

Figure 2 shows the crystallization and melting behaviors analyzed by DSC. Figure 2I shows that the crystallization onset temperatures of SSS-, SSO-, and OSO-rich fats were 46.63 °C, 21.73 °C, and 6.93 °C, respectively. The crystallization peaks of the SSS- and SSO-rich fats were distinct sharp at 44.96 °C and 20.68 °C, respectively, indicating that crystallization mostly occurs at these temperatures. In contrast, the OSO-rich fat had crystallization peaks at 5.20 °C and −16.81 °C in a relatively wide range from −20.61–6.93 °C, indicating two-step crystallization behavior because TAGs in the OSO-rich fat were more diverse than in the SSS-and SSO-rich fats (Table 4). 

The DSC melting curve (Figure 2II) showed that the SSS-rich fat contained higher melting TAGs than the SSO- and OSO-rich fats, as two distinct melting peaks were identified at 58.60 °C and 64.13 °C, and the fat started to melt at 53.19 °C and was completed at 66.45 °C. The SSO-rich fat contained TAGs with higher melting temperatures than the OSO rich fat, as indicated by the higher melting range of SSO-rich fat (16.83–34.28 °C) than OSO-rich fat (8.32–21.21 °C); although, the melting peaks of these fats were small and indistinct. Arishima et al. [17] reported that the melting ranges of SSS (purity 83%), PPP (purity 99%), and SSO (purity 99%) determined by DSC were 52.9–69.5 °C, 43.1–61.2 °C, and 29.5–42.6 °C, respectively, and OSO (purity 99%) was completely melted at 22.7 °C. The melting range was higher for the SSS-rich fat than PPP and lower than that of SSS, indicating that the TAGs of the SSS-rich fat comprised of a mixture of SSS, PSS, PSP, and PPP. The melting range of the SSO-rich fat was broader at lower temperatures than that of SSO (purity 99%) because the synthesized TAGs of the SSO-rich fat included additional lower melting TAGs such as OSO and SOO. 

### 2.4. Solid Fat Index (SFI)

**Figure 3:**
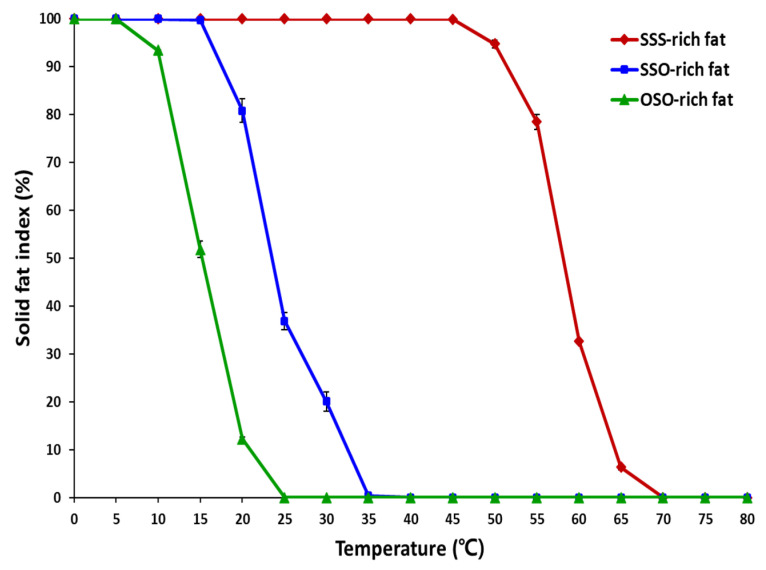
Solid fat index (SFI) of SSS-rich fat, SSO-rich fat, and OSO-rich fat.

Solid fat content (SFC) is very important physical property of lipids, which express the amount of solid fat at the measured temperature. The monitoring of SFC is essential to assess the melting profiles. Figure 3 showed that the SFI obtained by the DSC analysis, the SSO-rich and OSO-rich fats started to liquefy from the temperatures of 15 °C and 5 °C, respectively, and the SSO-rich fat contained more solid fats than the OSO-rich fat showing SFIs of 99.7% and 51.8%, respectively, at 15 °C, and SFIs of 80.8% and 12.24%, respectively, at 20 °C. The SSO-rich fat still contained solid fat having SFIs of 36.9% and 20.1%, respectively, at 25 °C and 30 °C, while OSO-rich fat was completely liquefied after 25 °C. In contrast, the SFI of SSS-rich fat were 94.7%, 75.4%, 32.6%, and 6.35%, respectively, at 50 °C, 55 °C, 60 °C, and 65 °C and this fat was completely liquefied after 70 °C. 

SFC is an important temperature dependent property of fats that determine their utilities in various application such as cocoa butter in chocolate and margarine in bakery products. Margarine is classified into bakery margarine (hard and medium plastic characteristic), and brick and tub margarines (medium plastic and soft characteristic). Brick type margarine has a SFC range of 47–60% at 5 °C, and 38–50% at 10 °C, and 19–26% at 20 °C, and such melting profile keep the brick shape at 20 °C, and is spreadable at refrigerator temperature [18]. Whereas, pastry margarine has a SFC range of 65–70% at 20 °C, and 30–40% at 40 °C with a flatter SFC profile than other margarines, and such SFC profile provide a long plasticity range which allows the bakery dough to be folded and rolled, and finally producing flaky texture in pastry products [18]. For the cocoa butter, the SFC was 76.4%, 67.2%, 41.5%, and 0.5% at 20 °C, 25 °C, 30 °C, and 35 °C, respectively, having very steep melting profile between 25 and 35 °C [19]. The SSO-rich and OSO-rich fats have steep melting profiles at 10–25 °C and 20–35 °C, respectively, and these fats can be used by blending with other fats and oils as a valuable plastic fat stock for providing proper melting profile in bakery process, and can be utilized in various food application as alternatives to *trans*-fat.

### 2.5. Crystal Microstructure 

**Figure 4:**
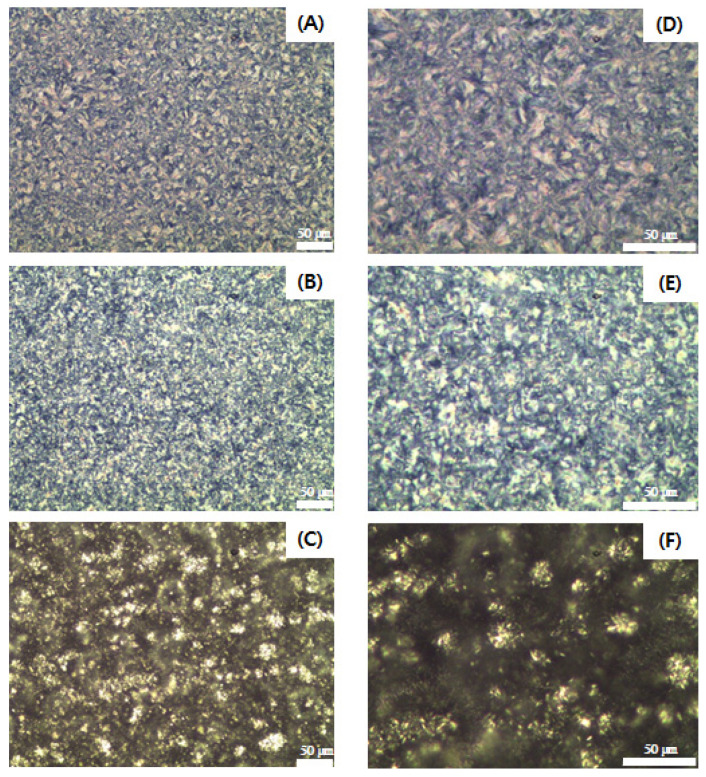
Polarized light micrographs of SSS-rich fat ((**A**): ×20, (**D**): ×40), SSO-rich fat ((**B**): ×20, (**E**): ×40), OSO-rich fat ((**C**): ×20, (**F**): ×40). The scale bar represents 50 μm.

Figure 4 illustrates the distinctly different microstructural morphologies of the SSS-, SSO-, and OSO-rich fats crystallized at 4 °C for 16 h. The microstructure of SSS-rich fat appears in the form of Maltese crosses, and the crystal morphology is similar to that of fully hydrogenated canola oil, which is composed of a mixture of SSS (79.6%) and PSS (16.3%) [20]. The SSO- and OSO-rich fats showed spherulite-shaped crystals that tended to aggregate to form clusters. Larger and more densely packed crystals were observed in the SSS-rich fat, followed by the SSO-rich and OSO-rich fats. According to the DSC crystallization curve (Figure 2I), the crystallization of SSS- and SSO-rich fat was completed at 40.88 °C and 14.91 °C, respectively, and the OSO-rich fat was crystallized by a two-step process at temperatures of −16.81 °C and 5.20 °C. Thus, the SSS-rich and SSO-rich fats were considered to have completed the crystallization at 4 °C, and their microstructures were larger and highly dense, whereas the OSO-rich fat had fewer diverse aggregates of clusters with small crystals and lower contents of solid fat holding the liquid oil inside the crystal networks since the crystallization process was still in progress.

### 2.6. Digestibility of SSO-Rich and OSO-Rich Fats Determined with In Vitro pH-Stat Model

**Figure 5:**
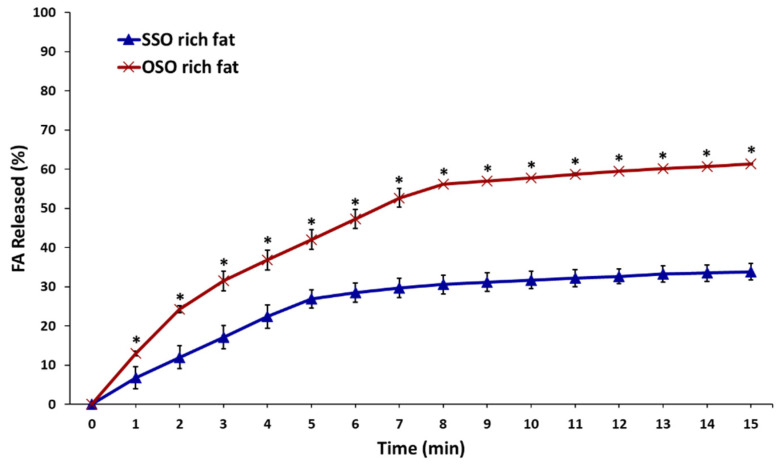
Free fatty acids released (%) of the SSO-rich fat and OSO-rich fat by in vitro pH-stat digestion model. * Mean on the graph are significantly different by Student’s *t*-test at *p* < 0.05.

The in vitro digestibility of the SSO-rich and OSO-rich fats was assessed under simulated small intestine conditions with pancreatin and pancreatic lipase in the gastric juice by the pH-stat method, and the amount of FFAs released (%) from TAGs over time from SSO-rich fat and OSO-rich fats was compared (Figure 5). The SSS-rich fat was excluded for the digestibility study because the CMP of 71.25 °C indicated that this fat was not completely dissolved at 37 °C, as the analytical temperature of pH-stat method. 

The released FFA (%) continuously increased with digestion until 15 min, and the final released FFA (%) from OSO-rich fat and SSO-rich fats were 61.4% and 33.8%, respectively, indicating a faster in vitro digestion of OSO-rich fat than SSO-rich fat (*p* < 0.05). These findings were apparently attributed to the composition of the FA at the *sn*-1,3 position of these fats. The fat digestion is affected by the composed FAs which have different stereospecificity (*sn*-1,3 or *sn*-2), and different chain lengths and degrees of saturation/unsaturation in TAG molecules [2]. TAGs are hydrolyzed by pancreatic lipase in the gastric juice, which has greater affinity for ester bonds in the *sn*-1 (or 3) position of TAG, resulting in the release of FFAs and 2-monoacylglycerol (2-MAG), in which most FAs at the *sn*-1 (or 3) positions are hydrolyzed [3]. Pancreatic lipase is more active against unsaturated FA than saturated FA [2]. The OSO-rich fat contained 1.4-fold more and 2.6-fold less unsaturated and saturated FAs, respectively, than the SSO-rich fat with TAGs at *sn*-1 (or 3) and OSO-rich and SSO rich-fats contained 1.43- and 4.1-fold more oleic and stearic acids, respectively (Table 2). The TAG in the small intestine was hydrolyzed by pancreatic lipase, which has greater affinity for ester bonds in the *sn*-1 (or 3) position of TAG, and it is more active against short-chain than long-chain FAs and against unsaturated than saturated FAs [2]. 

### 2.7. Digestibility of SSO-Rich and OSO-Rich Fats Determined Using In Vitro Multi-Step Digestion Model 

**Figure 6:**
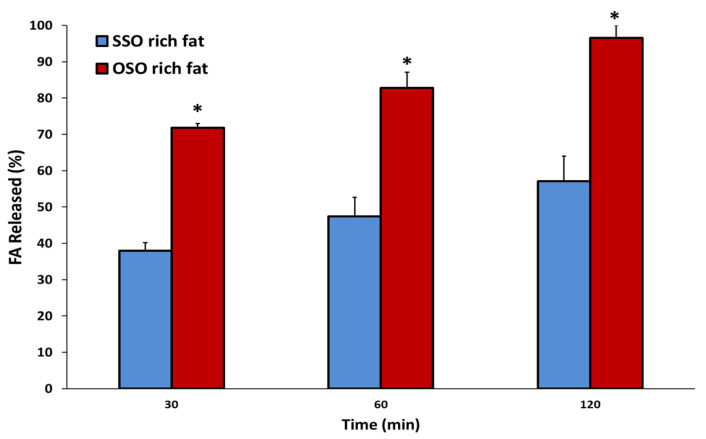
Free fatty acids released (%) of the SSO-rich and OSO-rich fats during in vitro multi-step digestion model (30, 60, 120 min). * Means on the bars in each digestion time are significantly different by Student’s *t*-test at *p* < 0.05. Mean ± SD (*n* = 2).

The in vitro multistep digestion rates of SSO- and OSO-rich fats were measured as released FFA (%) content, and the digestibility was evaluated based on the profiles of FAs released at 30, 60, and 120 min of digestion (Figure 6 and Table 5). The released FFA (%) of each fat significantly increased with as digestion proceeded. The released FFA (%) from OSO-rich fat was 71.8%, 82.8%, and 96.6%, at 30, 60, and 120 min of multi-step digestion, respectively, whereas those of SSO-rich fat were 37.9%, 47.4%, and 57.2%, respectively. Therefore, 1.89-, 1.75-, and 1.69-fold more FFA (%) was hydrolyzed in OSO- than in SSO-rich fat. In addition, the incorporated FAs at the *sn*-1,3 position of OSO-rich fat were almost completely hydrolyzed by in vitro digestion for 120 min. The authors previously showed that OPO-rich lipid was more easily digested than PPO-rich lipid, as the amounts of FFAs (%) released from each at 30, 60, and 120 min were 88.6%, 91.35%, and 104.9%, and 64.9%, 69.7%, and 75.7%, respectively, during the same in vitro multi-step digestion [21]. Therefore, the in vitro digestibility of lipids was affected by the type of FAs incorporated at the *sn*-1,3 position of TAG, and the digestibility was increased by less saturation and FAs with shorter carbon chains in the following descending order of OPO- > PPO- > OSO- > SSO-rich fats.

The position of FAs in TAG molecules also affects the extent of lipid absorption. A study of stearic-oleic TAGs in rats fed with calcium- and magnesium-deficient diets found that 99.5% and 70.9% of OSO and SSO, respectively were absorbed [22]. Oleic acid in the OSO diet was completely digested showing 99.9% absorption, and stearic acid was also well absorbed as 99.2%. In contrast, oleic and stearic acids were absorbed as 93.5% and 60.1%, respectively, in rats fed with an SSO diet. However, when rats were given SSO with a calcium- and magnesium-sufficient diet, only 59% of stearic acid was absorbed [22].

In the rats, OSO fed was completely hydrolyzed into oleic acid and 2-monostearin as digestion products, of which the stearic acid was apparently well absorbed since it was located at *sn*-2 position. The digestion products of SSO fed in the rats were oleic and stearic acids, and 2-monostearin, in which the intermediate hydrolyzed DAGs, 1-oleoyl-2-stearoylglycerol, and 1,2-disteain were formed. The 1-oleoyl-2-stearoylglycerol was then hydrolyzed to oleic acid and 2-monostearin which was absorbed, whereas 1,2-distearin would be not hydrolyzed because its high melting temperature (77.2 °C) might have mitigated micelle formation with emulsifiers [23,24]. The melting point and solubility of lipids during digestion significantly affect digestion [24].

### 2.8. Profiles of the Released FFAs of SSO-Rich and OSO-Rich Fats during In Vitro Multi-Step Digestion Model

The main FAs incorporated at the *sn*-1,3 position of the SSO-rich and OSO-rich fats were oleic (54.7% and 78.4%, respectively), stearic (32.1% and 7.8%, respectively), palmitic (7.5% and 6.4%, respectively), and linoleic acids (3.1% and 4.5%, respectively) (Table 2). During in vitro multi-step digestion, FFAs released from the hydrolyzed SSO-rich and OSO-rich fats were monitored at 30, 60, and 120 min, and Table 5 shows the individual FAs as ratios (%) of total hydrolyzed FAs. As digestion of the SSO-rich and OSO-rich fats was prolonged, the amounts of released unsaturated FAs tended to decrease (68.4%→67.6%→64.7%, and 81.2%→78.6%→76.9%, respectively), whereas the release of saturated FAs relatively increased (31.6%→32.4%→35.3%, and 18.8%→21.4%→23.1%, respectively). The *sn*-1,3 positions of SSO-rich fat contained 7.5% of palmitic acid and 54.7% of oleic acid, which accounted for 9.61% and 64.3% of the FFAs released after 30 min of digestion, whereas the stearic acid content of 32.1% accounted for 21.9% of the total released FAs (Table 5). That is, the initial digestion rate at 30 min was higher for palmitic and oleic acids than for stearic acid. However, after 60 and 120 min of digestion, the distribution of palmitic and oleic acids decreased to 8.3% and 7.9% and 63.6% and 60.8%, respectively, whereas that of stearic acid increased by 23.7% and 26.9%, respectively.

As shown in Figure 6, the SSO-rich fat was slowly hydrolyzed, releasing 37.93% FAs during the initial 30 min, and increased to 47.4% and 57.2% at 60 and 120 min of digestion, respectively. Whereas the hydrolysis rate of OSO-rich fat was higher than that of SSO-rich fat, by releasing 71.8% FAs at the initial 30 min digestion, which is 1.9 times higher (*p* < 0.05), and at the final 120 min digestion, 96.6% of FAs were released; among the FAs, oleic acid was the highest (72.29%), and followed by stearic acid (16.75%) and palmitic acid (5.72%). In addition, stearic acid (7.84% incorporated at *sn*-1,3) showed a relatively highly released rate (12.4%) at 30 min digestion, and then continuously increased to the final digestion (14.64%→16.75%), and palmitic acid (6.4% incorporated at *sn*-1,3) showed a similar released rate during digestion (6.0%→6.3%→5.7%). The reaction temperature in the multi-step digestion model performed was 37 °C, which is lower than the CMP (38.75 °C) of SSO-rich fat, so it is considered that the hydrolysis was somewhat difficult because high-melting TAGs having stearic acid were not sufficiently dissolved during digestion process. Because the OSO-rich fat with relatively low CMP (24.25 °C) was completely dissolved during the digestion process, the lipase hydrolyzed the TAG well, and in the initial 30 min digestion, not only oleic acid, but also stearic and palmitic acids, which are relatively difficult to be hydrolyzed, were hydrolyzed well simultaneously. 

The initial hydrolytic products of SSO-rich fat during digestion were mainly oleic acid and 2,3-distearoylglycerol. During digestion of SSS-rich fat, the SSO became oriented to the surface of the fat particles in which the 1-oleoyl group were aqueous facing and the 2,3-distearoyl group were lipid facing, so that the *sn*-1,3 regiospecific lipase hydrolyzed the 1-oleoyl group, but remained a 2,3-distearoyl group in the fat particle from where it was difficult for 2,3-distearoylglycerol to be hydrolyzed by pancreatic lipase [23] since the lipases are water soluble, and acts at the interface of fat and aqueous mixtures [25]. The present study showed that oleic acid was digested more rapidly than stearic acid during the initial digestion, resulting in a rapid increase in the amount of FFA (%) released relative to total hydrolyzed FAs. Previous studies regarding TAGs with a high amount of stearic acid were carried out invidiously for manufacturing [13], TAG and FA profile, evaluation of melting and crystallization property [12], and absorption [23]. In the present study, the synthesis of stearoyl-richer fats, and the evaluation of physicochemical properties followed by assessment of digestion property, were consequently evaluated. It was found that the incorporated location and amount of stearic acid in TAG molecules affected melting and crystallization, and whose properties consequently influenced digestion behavior, resulting in a difficult-to-digest-fat, which is valuable property in food lipid industry.

## 3. Materials and Methods

### 3.1. Materials

FHSBO was provided from CJ Cheil Jedang Co., Ltd. (Seoul, Korea). Oleic acid, bile salts, pepsin from porcine gastric mucosa, lipase from porcine pancreas, bovine serum albumin (BSA), pancreatin from porcine pancreas, mucin from porcine stomach, and tetramethylsilane (TMS) were purchased from Sigma-Aldrich Co., Ltd. (St. Louis, MO, USA). Chloroform-D1(CDCl_3_) was obtained from Merck group (Darmstadt, Germany). The immobilized lipase Lipozyme^®^ RMIM was purchased from Novo Nordisk Inc. (Plainsboro, NJ, USA).

### 3.2. Synthesis of SSO-Rich and SOS-Rich Fats 

**Figure 7:**
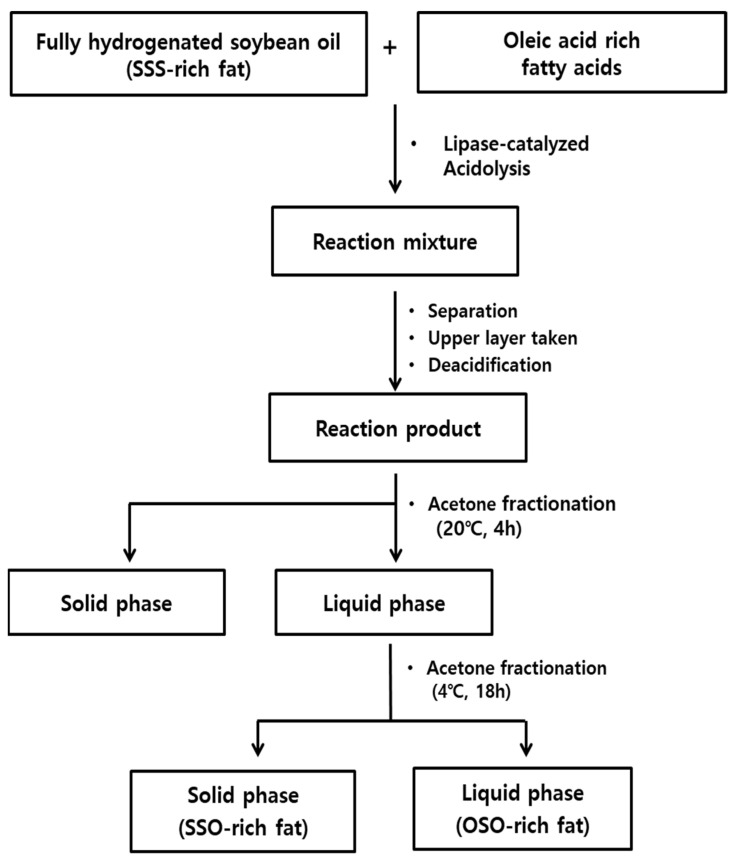
Production scheme of SSO-rich and OSO-rich fats.

SSO-rich and OSO-rich fats were synthesized from FHSBO (SSS-rich fat) and oleic acid rich fatty acids by lipase-catalyzed acidolysis followed by acetone fractionation, as presented in Figure 7 [21]. The completely dissolved FHSBO was mixed with oleic acid (1:2 molar ratio) in an Erlenmeyer flask (500 mL) with screw cap. Lipozyme^®^ RMIM (10%, *w*/*w*) and *n*-hexane (50 mL) were added and lipase-catalyzed acidolysis reaction was carried out in a shaking water bath at 200 rpm and 55 °C for 6 h. After reaction, the reactant was mixed with *n*-hexane (1:4, *w*/*v*), and placed in 20 °C for 1 h to separate unreacted FHSBO (high-melting TAG). The upper layer of *n*-hexane was collected, deacidified with 2 N KOH in ethanol, concentrated using a rotary evaporator (N-1110, Sunileyela, Seongnam-city, Korea), and dried completely with N_2_. The deacidified reactant was completely dissolved at 80 °C, and mixed with acetone (1:4, *w*/*v*) and fractionated at 20 °C for 4 h. The filtrated upper liquid phase was placed at 4 °C for 18 h, and fractionated into the solid phase (SSO-rich fat) and liquid phase (OSO-rich fat), and then dried with N_2_. For purification of TAG, each fractionated fat from liquid and solid phase was mixed with methanol (100 mL) for 30 min, and methanol layer was discarded (repeat 6 times), and dried with N_2_.

### 3.3. Acylglycerol Composition

The fat (70 mg) was dissolved in 0.1 TMS in CDCl_3_ (700 μL), and placed in a 5 mm NMR tube (Norell, Landisville, NJ, USA). The acylglycerol composition was analyzed with Bruker Advance III 600 MHz NMR spectrometer (Bruker Corporation, Billerica, MA, USA). ^1^H-NMR spectra were obtained, and the chemical shifts of signals of TAG (5.255–5.283 ppm), 1,2-DAG (5.065–5.107 ppm), 1,3-DAG (4.054–4.099 ppm), 2-MAG (4.911–4.947 ppm), and 1-MAG (3.902–3.947 ppm) were identified and quantified with tetramethylsilane (TMS) as a reference, and expressed in mmol% units [26]. 

### 3.4. TAG Composition 

The TAG composition was analyzed with Ag-HPLC (Younglin, Anyang, Korea) equipped with a silver ion column of ChromSpher 5 lipids (pore size: 120 Å, particle size: 5.0 μm, 250 mm × 4.6 mm i.d., Varian, Middleburg, Netherlands) and evaporative light scattering detector (ELSD, Sedex 75, Sedere, Alfortville, France). The mobile phases were solvent A (*n*-hexane: acetonitrile: iso-propanol = 100:0.1:0.1, *v*/*v*/*v*) and solvent B (*n*-hexane: acetonitrile: iso-propanol = 100:1:1, *v*/*v*/*v*). The solvent gradient was started at a ratio of 100:0 (A:B, *v*/*v*) for 5 min, and changed to 80:20 for 45 min, to 50:50 for 10 min, and held for 1 min, then returned to the initial ratio, and finally held for 8 min. The fats were dissolved in chloroform: *n*-hexane (1:1, *v*/*v*), and the injection volume was 20 μL. The temperature of ELSD and flow rate was set at 40 °C and 1.5 mL/min, respectively, and N_2_ was used as a nebulizing gas at a pressure of 2.2 bar. The TAGs were separated with the number of double bond and the position (*sn*-1(or 3), *sn*-2) of fatty acids. In the obtained chromatogram of Figure 1, TAG was designated with saturated fatty acid (Sa), monounsaturated fatty acid (Mo), and diunsaturated fatty acid (D). 

### 3.5. FA Composition

The FA composition was analyzed with gas chromatography (GC-2010 Plus, Shimadzu Corp., Kyoto, Japan) equipped with a flame ionization detector and SP^TM^-2560 capillary column (100 m × 0.25 mm × 0.2 μm film thickness, Supelco Inc., Bellefonte, PA, USA). The fat (25 mg) was saponified with 0.5 N NaOH in methanol, and methylated with 1.5 M BF_3_ in methanol. The temperature of GC oven was held at 100 °C for 5 min, increased to 240 °C at the rate of 4 °C /min, and maintained for 40 min. The temperatures of injector and detector were 250 °C and 260 °C, respectively. The split ratio was 100:1, the flow rate of carrier gas was 1.00 mL/min (N_2_), and the injection volume was 1 μL. 

For positional FA composition, fat (5~10 mg), pancreatic lipase (5~10 mg), Tris-HCl buffer (pH 7.6, 5 mL), 2.2% CaCl_2_ (0.5 mL), and 0.05% bile salt (1.25 mL) were mixed in a test tube, and reacted for 3 min at 37 °C with 30 s vortexing, and repeated 5 times. Then, diethyl ether (6 mL) was added and mixed for 1 min. After centrifugated (1224× *g*, 5 min), the supernatant was concentrated with N_2_, and separated on a thin-layer chromatography (TLC) F_254_ silica plate (20 × 20 cm, Merck, Kenilworth, NJ, USA) with a developing solvent (*n*-hexane: diethyl ether: acetic acid = 50:50:1, *v*/*v*/*v*). The 2-MAG band was taken from the TLC plate, after saponification and methylation, the positional fatty acids were analyzed with GC, and calculated with the following equation:

FA composition at *sn*-1,3 position (%) = [3 × total FA composition (%) − FA composition at *sn*-2(%)]/2

### 3.6. Melting and Crystallization Behaviors and SFI 

The melting point was analyzed with capillary tube method of AOCS Official Method Cc 1–25 (AOCS, 2009) [27]. The completely melted fat was filled into 1 cm height of capillary tube (internal diameter of 1 mm), and tempered below −20 °C for 16 h. The tube was heated in CuSo_4_·5H_2_O solution in a beaker, starting from 0 °C at a rate of 0.5 °C/min. The SMP is defined as the temperate at which the fat begins to rise in the tube, and the (CMP is defined as the temperature at which the fat becomes completely clear and liquid. The melting and crystallization behaviors were analyzed with differential scanning calorimetry (DSC-8000, Perkin Elmer, Waltham, MA, USA). The completely melted fat (1~3 mg) in DSC aluminum pan was heated to 80 °C and held for 10 min. DSC crystallization thermogram was obtained by cooling to −60 °C at 10 °C/min, then holding for 10 min, and the melting thermogram was obtained by heating to 80 °C at 5 °C/min. The SFI (%) was determined by using the DSC melting thermogram, and the SFI at a given temperature was calculated at the ratio of the area resulting from the integration of peaks from the thermogram. 

### 3.7. Crystal Morphology 

The completely melted fat (20 μL) at >80 °C was placed on a preheated microscope glass slide, and covered with preheated cover slip. The prepared slide was stored at 4 °C for 16 h, and the fat crystal morphology was obtained by polarized light microscopy (Eclipase 50i POL, Nikon, Tokyo, Japan) equipped with digital camera (ISH 300, Tucsen Photonics Co., Ltd., Fuzhou, China). 

### 3.8. In Vitro Digestion with pH-Stat Model

In vitro digestion of SSO-rich and OSO-rich fat was performed with a pH-Stat model under simulated small intestine conditions of the modified method of Versantvoort et al. [28] and Ji et al. [29]. Digestion juice was freshly prepared by mixing duodenal juice and bile juice (2:1, *v*/*v*), and enzyme solution was made by dissolving pancreatin (54 mg) and lipase (36 mg) in the digestion juice (1 mL). Each fat (500 mg) was dispersed into digestion juice (35 mL) in a 100 mL beaker by an ultrasonic processor (VC750, Sonics & Materials Inc., Newtown, CT, USA) for 1 min. The hydrolysis of fat was started by adding enzyme solution (1 mL) into the digestive juice in a water bath at 37 °C and 150 rpm for 15 min. During hydrolysis, the released FFAs was measured by a titration with a 50 mN NaOH solution, and the pH of the titration was automatically maintained at pH 8.1 with a potentiometric automatic titration (AT-400E, Kyoto Electronics Manufacturing Co., Tokyo, Japan). The added volume of 50 mM NaOH solution was recorded at 1 min intervals, and the released FFA percentage from the fat was calculated using the following equation;

$$\mathrm{The}\;\mathrm{released}\;\mathrm{FFA}\;\left(\%\right)=\frac{\mathrm{Vollume}\;\mathrm{of}\;\mathrm{NaOH}\;\left(\mathrm{mL}\right)\times \;\mathrm{Normality}\;\mathrm{of}\;\mathrm{NaOH}\;\left(\mathrm{mM}\right)}{\mathrm{Total}\;\mathrm{amount}\;\mathrm{of}\;\mathrm{digestible}\;\mathrm{FFA}\;\left(\mu \mathrm{mol}\right)}\times 100$$

### 3.9. In Vitro Multi-Step Digestion Model

In vitro digestion rates of SSO-rich and OSO-rich fats were determined under simulated mouth, stomach, and small intestine digestive condition of the modified method of Versantvoort et al. [28] and Chang and Lee [21]. The melted fat (100 mg) and saliva juice (1.2 mL) were mixed in a 100 mL Erlenmeyer flask in shaking water bath at 37 °C and 80 rpm for 5 min, and gastric juice (2.4 mL) was added and reacted for 2 h. Bile juice (1.2 mL), duodenal juice (2.4 mL), and NaHCO_3_ solution (0.4 mL) were added, and the digestion of fat proceeded for 30, 60, and 120 min. After which, the enzyme (lipase) was inactivated by adding an inhibitor (100 μL, 0.2 g of 4-bromophenylboronic acid/mL in methanol), and the digestion was stopped. For extracting of digested fat, *n*-hexane was mixed and centrifuged (1763× *g*; 5 min), and the supernatant was taken (repeated 3 times). The remained lower part was mixed with 1 N HCl (0.5 mL) for 1 min. After mixing with *n*-hexane (10 mL) and centrifugation, the supernatant was collected (repeated 3 times), combined and concentrated with N_2_. The digested fat was mixed with ethanol:*n*-hexane (1:1, *v*/*v*; 10 mL) and 1% phenolphthalein (1 mL), and the FFAs were titrated with 50 mM KOH solution. The extent of in vitro digestion of SSO- and OSO-rich fats was expressed as released FFAs (%) using the same calculation formula as the pH-Stat model. For analysis of the released fatty acid composition during in vitro multi-step digestion, each hydrolyzed fat at 30, 60, and 120 min of digestion was extracted and separated on TLC plate with developing solvent (*n*-hexane: diethyl ether: acetic acid = 50:50:1, *v*/*v*/*v*). The FFA band was taken and methylated, and the fatty acid composition was analyzed with GC.

### 3.10. Statistical Analysis

Analysis of variance (ANOVA) was performed using the Statistical Analysis System 9.4 (SAS Institute, Cary, NC, USA). The statistical significance of experimental means was determined by Student’s *t*-test or Duncan’s multiple range test at *p* < 0.05.

## 4. Conclusions

Stearic acid has the essential physical attributes of solid fats, and stearoyl-rich fat is a viable substitute for TFAs in food manufacturing. Specifically modified stearoyl-rich TAG-rich (SSO- and OSO-rich) fats with different contents and rearranged regiospecific positions of stearic acid were synthesized via a lipase-catalyzed acidolysis between SSS-rich and oleic acids and solvent fractionation. Their physicochemical properties and in vitro digestibility were compared. The SSS-, SSO-, and OSO-rich fats mainly contained SaSaSa, SaSaMo, and MoSaMo of TAGs, respectively, and their major TAGs were SSS, SSO, and OSO, respectively. Stearic acid content, melting point, crystallization temperature, solid fat index, and packing density of fat crystals decreased in the following order: SSS- > SSO- > OSO-rich fats. The OSO-rich fat was digested more rapidly than the SSO-rich fat. Among the FAs at the *sn*-1,3 position of TAGs, oleic acid was initially digested faster than stearic acid, then this slowed during further digestion, while the digestion rate of stearic acid increased. Fats containing stearoyl-richer TAGs melted at a higher temperature, were not very digestible in vitro, and comprised a densely-packed microstructure of large fat crystals. These stearoyl-richer TAGs should be considered for a use as a valuable difficult-to-digest fat in the food lipid industry. Further studies are needed to confirm the results of the stearoyl-richer fats by assessing whether the higher content and regiospecificity of stearic acid affect digestibility in in vivo animal models.

## Figures and Tables

**Table 1:** The total fatty acids composition of the fats.

Fatty Acid (% of Total Fatty Acids)	SSS-Rich Fat ^(1)^	SSO-Rich Fat	OSO-Rich Fat	Oleic Acid Rich Fatty Acids
C12:0	0.84 ± 0.02 ^a (2)^	0.55 ± 0.03 ^b^	0.80 ± 0.04 ^a^	- ^(3)^
C16:0	13.09 ± 0.07 ^a^	5.89 ± 0.08 ^b^	5.26 ± 0.06 ^c^	0.87 ± 0.01 ^d^
C18:0	81.63 ± 0.03 ^a^	52.89 ± 0.15 ^b^	33.14 ± 0.11 ^c^	2.41 ± 0.01 ^d^
C18:1*t*	-	0.68 ± 0.00 ^c^	0.89 ± 0.00 ^b^	2.06 ± 0.00 ^a^
C18:1n-9*c*	2.86 ± 0.05 ^d^	37.45 ± 0.04 ^c^	56.23 ± 0.01 ^b^	90.53 ± 0.01 ^a^
C18:2n-6*c*	0.64 ± 0.01 ^d^	2.06 ± 0.01 ^c^	3.44 ± 0.03 ^b^	4.08 ± 0.00 ^a^
C20:0	0.57 ± 0.00 ^a^	0.30 ± 0.00 ^b^	0.15 ± 0.00 ^c^	0.04 ± 0.00 ^d^
C22:0	0.34 ± 0.01 ^a^	0.19 ± 0.00 ^b^	0.10 ± 0.00 ^c^	-
ΣSFA ^(4)^	96.46 ± 0.06 ^a^	59.81 ± 0.05 ^b^	39.44 ± 0.02 ^c^	3.33 ± 0.00 ^d^
ΣUSFA ^(4)^	3.54 ± 0.06 ^d^	40.19 ± 0.05 ^c^	60.56 ± 0.02 ^b^	96.67 ± 0.00 ^a^
ΣMUFA ^(4)^	2.89 ± 0.05 ^d^	38.13 ± 0.04 ^c^	57.11 ± 0.01 ^b^	92.59 ± 0.00 ^a^
ΣPUFA ^(4)^	0.64 ± 0.01 ^d^	2.06 ± 0.01 ^c^	3.44 ± 0.03 ^b^	4.08 ± 0.00 ^a^
Melting Points				
Slip melting point (°C)	69.75 ± 1.06 ^a (2)^	32.50 ± 0.71 ^b^	19.75 ± 0.35 ^c^	
Complete melting point (°C)	71.25 ± 0.35 ^a^	38.75 ± 0.35 ^b^	24.25 ± 0.35 ^c^	

^(1)^ SSS-rich fat: Fully hydrogenated soybean oil; ^(2)^ Mean ± SD (*n* = 2). ^a–d^ Means in the same row with different letters are significantly different by Duncan’s multiple range test at *p* < 0.05; ^(3)^ Not Detected; ^(4)^ SFA: Saturated fatty acid, USFA: Unsaturated fatty acid, MUFA: Monounsaturated fatty acid, PUFA: Polyunsaturated fatty acid.

**Table 2:** The *sn*-2 and *sn*-1,3 positional fatty acids composition of the fats.

Fatty Acid (% of Total Fatty Acids)	*sn*-1,3 Position			*sn*-2 Position		
	SSS-Rich Fat ^(1)^	SSO-Rich Fat	OSO-Rich Fat	SSS-Rich Fat	SSO-Rich Fat	OSO-Rich Fat
C12:0	0.62 ± 0.08 ^b (2)^	0.82 ± 0.05 ^b^	1.21 ± 0.05 ^a^	1.25 ± 0.26	- ^(3)^	-
C16:0	17.24 ± 0.23 ^a^	7.50 ± 0.10 ^b^	6.40 ± 0.10 ^c^	4.75 ± 0.22 ^a^	2.65 ± 0.03 ^b^	2.97 ± 0.04 ^b^
C18:0	80.66 ± 0.46 ^a^	32.14 ± 0.34 ^b^	7.84 ± 0.19 ^c^	82.69 ± 1.02 ^b^	94.39 ± 0.02 ^a^	83.74 ± 0.05 ^b^
C18:1*t*	-	1.02 ± 0.00 ^b^	1.33 ± 0.01 ^a^	-	-	-
C18:1n-9*c*	-	54.70 ± 0.18 ^b^	78.36 ± 0.02 ^a^	9.64 ± 0.77 ^b^	2.96 ± 0.25 ^c^	11.96 ± 0.01 ^a^
C18:2n-6*c*	0.12 ± 0.11 ^c^	3.08 ± 0.02 ^b^	4.49 ± 0.05 ^a^	1.66 ± 0.21 ^a^	-	1.34 ± 0.02 ^a^
C20:0	0.85 ± 0.01 ^a^	0.45 ± 0.01 ^b^	0.22 ± 0.00 ^c^	-	-	-
C22:0	0.51 ± 0.01 ^a^	0.28 ± 0.01 ^b^	0.14 ± 0.00 ^c^	-	-	-
ΣSFA ^(4)^	99.88 ± 0.56 ^a^	41.20 ± 0.20 ^b^	15.81 ± 0.03 ^c^	88.69 ± 0.98 ^b^	97.04 ± 0.25 ^a^	86.70 ± 0.01 ^c^
ΣUSFA ^(4)^	0.12 ± 0.11 ^c^	58.80 ± 0.20 ^b^	84.19 ± 0.03 ^a^	11.31 ± 0.98 ^b^	2.96 ± 0.25 ^c^	13.30 ± 0.0 1^a^
ΣMUFA ^(4)^	-	55.72 ± 0.18 ^b^	79.69 ± 0.01 ^a^	9.64 ± 0.77 ^b^	2.96 ± 0.25 ^c^	11.96 ± 0.01 ^a^
ΣPUFA ^(4)^	0.12 ± 0.11 ^c^	3.08 ± 0.02 ^b^	4.49 ± 0.05 ^a^	1.66 ± 0.21 ^a^	-	1.34 ± 0.02 ^a^

^(1)^ SSS-rich fat: Fully hydrogenated soybean oil; ^(2)^ Mean ± SD (*n* = 2). ^a–c^ Means in the same row with different letters are significantly different by Duncan’s multiple range test at *p* < 0.05; ^(3)^ Not Detected; ^(4)^ SFA: Saturated fatty acid, USFA: Unsaturated fatty acid, MUFA: Monounsaturated fatty acid, PUFA: Polyunsaturated fatty acid.

**Table 3:** The acylglycerol composition of the fats.

Acylglycerol (mmol%)	SSS-Rich Fat ^(1)^	SSO-Rich Fat	OSO-Rich Fat
Triacylglycerol (TAG)	98.31 ± 0.69 ^a (2)^	94.68 ± 1.03 ^b^	98.58 ± 0.14 ^a^
Diacylglycerol (DAG)	1.38 ± 0.75 ^b^	4.78 ± 0.59 ^a^	0.97 ± 0.04 ^b^
1,3-DAG	1.01 ± 0.66 ^b^	3.65 ± 0.79 ^a^	0.55 ± 0.21 ^b^
1,2-DAG	0.37 ± 0.09 ^b^	1.13 ± 0.20 ^a^	0.42 ± 0.25 ^b^
Monoacylglycerol (MAG)	0.31 ± 0.06 ^a^	0.53 ± 0.44 ^a^	0.45 ± 0.11 ^a^
1-MAG	0.17 ± 0.03 ^a^	0.33 ± 0.17 ^a^	0.24 ± 0.07 ^a^
2-MAG	0.14 ± 0.03 ^a^	0.21 ± 0.26 ^a^	0.20 ± 0.04 ^a^

^(1)^ SSS rich fat: Fully hydrogenated soybean oil; ^(2)^ Mean ± SD (*n* = 2); ^a,b^ Means in the same row with different letters are significantly different by Duncan’s multiple range test at *p* < 0.05.

**Table 4:** The triacylglycerol composition of SSS-rich, SSO-rich, and OSO-rich fats.

Triacylglycerol (% of Total TAGs)	SSS-Rich Fat	SSO-Rich Fat	OSO-Rich Fat
SaSaSa ^(1)^ (SSS)	100.00 ± 0.00 * ^(2)^	0.49 ± 0.05	- ^(3)^
SaMoSa	-	1.17 ± 0.08	1.34 ± 0.05
SaSaMo (SSO)	-	86.98 ± 0.13 *	17.22 ± 0.46
SaDSa	-	0.93 ± 0.09 *	0.32 ± 0.10
SaSaD/SaMoMo	-	1.22 ± 0.01 *	8.55 ± 0.73
MoSaMo (OSO)	-	9.21 ± 0.07 *	67.17 ± 1.16
SaDMo/SaMoD	-	-	0.45 ± 0.07
MoSaD	-	-	2.00 ± 0.02
MoMoMo	-	-	2.96 ± 0.10
Total	100.00	100.00	100.00

^(1)^ The abbreviations were same as presented in Figure 1; The abbreviations in parentheses refer to the major TAGs; ^(2)^ Mean ± SD (*n* = 2); * Mean in the same row are significantly different by Student’s *t*-test at *p* < 0.05 ^(3)^ -: Not Detected.

**Table 5:** The released fatty acids composition of the hydrolyzed SSO-rich and OSO-rich fats during in vitro multi-step digestion.

Fatty Acid (% of Total Fatty Acids)	SSO-Rich Fat			OSO-Rich Fat		
	30 Min	60 Min	120 Min	30 Min	60 Min	120 Min
C12:0	- ^(1)^	0.45 ± 0.04 ^a (2) (3)^	0.51 ± 0.04 ^a^	0.43 ± 0.06 ^b^	0.48 ± 0.04 ^b^	0.59 ± 0.05 ^a^
C16:0	9.61 ± 0.95 ^a^	8.25 ± 0.42 ^b^	7.92 ± 0.16 ^b^	6.00 ± 0.24 ^a^	6.25 ± 0.69 ^a^	5.72 ± 0.23 ^a^
C18:0	21.96 ± 2.31 ^a^	23.68 ± 1.06 ^a b^	26.90 ± 2.42 ^a^	12.40 ± 1.09 ^b^	14.64 ± 1.53 ^a b^	16.75 ± 1.80 ^a^
C18:1*t*	0.83 ± 0.04 ^a^	0.85 ± 0.05 ^a^	0.86 ± 0.02 ^a^	1.02 ± 0.03 ^a^	1.01 ± 0.03 ^a^	0.97 ± 0.03 ^a^
C18:1n-9*c*	64.30 ± 3.02 ^a^	63.60 ± 0.82 ^a^	60.82 ± 2.29 ^a^	76.49 ± 1.38 ^a^	73.99 ± 2.01 ^a b^	72.29 ± 1.83 ^b^
C18:2n-6*c*	3.30 ± 0.18 ^a^	3.17 ± 0.14 ^a b^	2.98 ± 0.13 ^b^	3.65 ± 0.05 ^a^	3.62 ± 0.11 ^a^	3.68 ± 0.14 ^a^
C20:0	-	-	-	-	-	-
C22:0	-	-	-	-	-	-
ΣSFA ^(4)^	31.58 ± 3.15 ^a^	32.38 ± 0.98 ^a^	35.34 ± 2.44 ^a^	18.83 ± 1.35 ^b^	21.37 ± 2.05 ^a b^	23.06 ± 1.97 ^a^
ΣUSFA ^(4)^	68.42 ± 3.15 ^a^	67.62 ± 0.98 ^a^	64.66 ± 2.44 ^a^	81.17 ± 1.35 ^a^	78.63 ± 2.05 ^a b^	76.94 ± 1.97 ^b^
ΣMUFA ^(4)^	65.12 ± 3.02 ^a^	64.45 ± 0.86 ^a^	61.68 ± 2.31 ^a^	77.51 ± 1.39 ^a^	75.01 ± 2.01 ^a b^	73.26 ± 1.85 ^b^
ΣPUFA ^(4)^	3.30 ± 0.18 ^a^	3.17 ± 0.14 ^a b^	2.98 ± 0.13 ^a^	3.65 ± 0.05 ^a^	3.62 ± 0.11 ^a^	3.68 ± 0.14 ^a^
Released FFA content (%)	37.93 ± 2.20 ^b^	47.39 ± 5.34 ^b^	57.15 ± 6.89 ^b^	71.76 ± 1.25 ^a^	82.76 ± 4.35 ^a^	96.58 ± 3.49 ^a^

^(1)^ -: Not Detected; ^(2)^ Mean ± SD; ^(3) a,b^ Means in the same row with different letters are significantly different at *p* < 0.05 by Duncan’s multiple range test; ^(4)^ SFA: Saturated fatty acid, USFA: Unsaturated fatty acid, MUFA: Monounsaturated fatty acid, PUFA: Polyunsaturated fatty acid.

## Data Availability

Not applicable.
